# Systematic review of the effect of cerebrospinal fluid drainage on outcomes after endovascular type B aortic dissection repair

**DOI:** 10.1186/s13019-024-02603-3

**Published:** 2024-03-12

**Authors:** Huajie Zheng, Deqing Lin, Yongbo Cheng, Chaojun Yan, Sanjiu Yu, Jun Li, Wei Cheng

**Affiliations:** Department of Cardiac Surgery, Southwest Hospital, Third Military Medical University (Army Medical University, No. 30, Gaotanyan Road, Shapingba District, Chongqing, 400038 P.R. China

**Keywords:** Aortic dissection, Thoracic endovascular aortic repair, Spinal cord ischemia, Cerebrospinal fluid drainage, Systematic review

## Abstract

**Objective:**

The aim of the present systematic review was to determine whether prophylactic use of cerebrospinal fluid drainage (CSFD) contributes to a lower rate of spinal cord ischemia (SCI) after thoracic endovascular aortic repair (TEVAR) for type B aortic dissection (TBAD).

**Methods:**

PubMed, Embase, Web of Science and Cochrane Library databases were systematically searched to identify all relevant studies reported before May 7, 2023. A systematic review was conducted in accordance with PRISMA guidelines (PROSPERO registration no. CRD42023441392). The primary outcome was permanent SCI. Secondary outcomes were temporary SCI and 30-day/in-hospital mortality. The data were presented as the pooled event rates (ERs) and 95% confidence intervals (CIs).

**Results:**

A total of 1008 studies were screened, of which 34 studies with 2749 patients were included in the present analysis. The mean Downs and Black quality assessment score was 8.71 (range, 5–12). The pooled rate of permanent SCI with prophylactic CSFD was identical to that without prophylactic CSFD (2.0%; 95% CI, 1.0–3.0; *P* = 0.445). No statistically significant difference was found between the rates of permanent SCI with routine vs. selective prophylactic CSFD (*P* = 0.596). The pooled rate of temporary SCI was 1.0% (95% CI, 0.00–1.0%). The pooled rate for 30-day or in-hospital mortality was not significantly different (*P* = 0.525) in patients with prophylactic CSFD (4.0, 95% CI 2.0–6.0) or without prophylactic CSFD (5.0, 95% CI 2.0–7.0).

**Conclusions:**

The systematic review has shown that prophylactic CSFD was not associated with a lower rate of permanent SCI and 30-day or in-hospital mortality after TEVAR for TBAD.

**Supplementary Information:**

The online version contains supplementary material available at 10.1186/s13019-024-02603-3.

## Introduction

Thoracic endovascular aortic repair (TEVAR) has been adopted as the first-line treatment for type B aortic dissection (TBAD) because of its lower mortality and postoperative complication rates compared with open surgical repair [1, 2]. Despite these advancements, postoperative spinal cord ischemia (SCI) with its catastrophic sequelae of paraplegia and paraparesis occurring in 2.5–8%, has remained a major concern [3]. Some clinical studies have suggested that prophylactic cerebrospinal fluid drainage (CSFD), which refers to drainage performed preoperatively in all patients (routine) or only in high-risk patients (selective), might decrease the postoperative risk of SCI after TEVAR [4]. However, the use of prophylactic CSFD has been debated in reported studies [5–7]. Some investigators have suggested the routine use of prophylactic CSFD for all patients undergoing TEVAR [8]. In contrast, others have preferred the selective use of prophylactic CSFD for patients at high risk of SCI, including those with left subclavian or internal iliac artery coverage, thoracic aortic coverage ≥ 20 cm long, and/or a history of abdominal aortic repair [9–11].

Previous systematic reviews and meta-analyses have been limited to specific approaches (open or endovascular; endovascular or medical management) [12–14], pathology (complicated or uncomplicated) [15], or assessment of intentional celiac artery coverage [16, 17]. There has been no inclusive contemporary analysis of the effect of CSFD on SCI after endovascular repair of TBAD. The objective of the present study was to determine whether prophylactic use of CSFD contributes to a lower rate of permanent SCI after endovascular repair of TBAD.

## Methods

### Protocol and registration

The present systematic review was performed according to the Preferred Reporting Items for Systematic Reviews and Meta-Analyses (PRISMA) statement and recommended guidelines [18]. The study protocol was registered with the International Prospective Register of Systematic Reviews (PROSPERO), #CRD42023441392.

### Literature source and search strategy

We systematically searched the PubMed, Embase, Web of Science and Cochrane Library databases for all potential studies with no restrictions on publication languages. The search was conducted on May 7, 2023 and included only reported data. We also manually searched the reference lists of the eligible studies and previous reviews to identify additional evaluable articles. The following MeSH (medical subject headings) terms or keywords were used: “aortic dissection” AND “stents” OR “stent graft” OR “endovascular” AND “spinal cord ischemia” OR “paraparesis” OR “paraplegia”. Details of the search strategy are reported in the Supplementary Appendix.

### Selection criteria

Two authors (Huajie Zheng and Deqing Lin) independently performed the literature search. They independently reviewed the titles and abstracts of all citations to identify potentially relevant studies and exclude any duplicates. They reviewed the full text of the corresponding reports to assess whether the studies had met the inclusion criteria. The references from these articles were also analyzed.

Studies were included if they had met the following criteria: (I) case-control study, cohort study, case series or randomized clinical trial; (II) studies reporting SCI rates (permanent or temporary) after TEVAR (elective or emergency) for TBAD; (III) studies reporting on routine prophylactic CSFD, selective prophylactic CSFD for high-risk patients, and no prophylactic CSFD. Studies were excluded if they had (I) not reported the TEVAR technique for TBAD; (II) not reported postoperative SCI rates; (III) not reported whether prophylactic CSFD or CSFD on demand (rescue drainage) had been used to treat SCI; (IV) overlapped with other reports of the same group (in such cases, the most recent report or the report with more details useful for the systematic review was included). The final inclusion of the studies was based on agreement between the reviewers. Any disagreement was resolved by discussion and consultation with the other coauthors (Chaojun Yan and Yongbo Cheng).

### Data extraction

A data extraction form was designed to collect all the variables from the eligible studies. The following data were extracted: first author’s name, publication year, study type, study period, location (where the study was undertaken), CSFD policy, indications for selective CSFD, CSFD duration, other neuroprotection methods, total patients, number of total SCI patients, number of permanent SCI patients, number of temporary SCI patients, 30-day/in-hospital mortality, and CSFD-related complications. The CSFD-related complications included epidural and intradural hematoma, catheter fracture, meningitis, intracranial hypotension, as well as post-lumbar puncture headache [19].

### Quality assessment

The quality of the included studies was independently scored by two authors (Huajie Zheng and Deqing Lin) in accordance with the Downs and Black score using 27 criteria to assess the quality of nonrandomized studies in terms of five quality domains (i.e., reporting, external validity, bias, confounding, and power) [20]. The total scores varied from 0 (poor quality) to 31 (high quality). Any disagreement was resolved by discussion with the other coauthors (Chaojun Yan and Yongbo Cheng).

### Outcome measures

The primary outcome was permanent SCI. Secondary outcomes were postoperative temporary SCI, and 30-day/in-hospital mortality. Permanent SCI was defined as any new onset of neurological symptoms of the lower extremities (paraparesis or paraplegia) following TEVAR, not caused by cerebral pathology. Only the complete resolution of SCI symptoms was not considered permanent SCI. The use of prophylactic CSFD was classified as routine (drain placed before intervention for patients considered at high risk of SCI) or selective (drain placed postoperatively in case of SCI).

### Statistical analysis

Outcomes were pooled as event rates (ERs) with 95% confidence intervals (CIs) using the generic inverse variance method [21, 22]. ERs were extracted from the individual studies or calculated based on the proportion of patients with the corresponding outcome among all patients treated. The pooled ERs and corresponding 95% CIs were estimated using fixed or random effect methods. Statistical heterogeneity was assessed by calculating the Cochran Q (χ^2^), calculated as the weighted sum of squared differences between individual study effect sizes and the overall pooled effect estimate, and its corresponding *P* value and *I*^*2*^ across the studies [23]. Heterogeneity was considered statistically significant at *P* < 0.05 and *I*^*2*^ > 50% for all measures. Sensitivity analyses were performed for all outcome measures to examine the robustness of the pooled estimates by removing one study at a time and recalculating the pooled effects. The results from the sensitivity analyses were considered statistically significant when the corrected estimates were beyond the 95% CI of the original estimates. Subgroup analyses were performed to assess the heterogeneity of the association between CSFD strategies and SCI rates after TEVAR. Publication bias was assessed using the Egger test and visual inspection of funnel plots of standard error against effect size. Asymmetry in the funnel plots, and *P* < 0.05 in the Egger test implied the existence of a publication bias. The outcomes reported in > 10 studies were used to assess the publication bias. All statistical analyses were performed using STATA software (version 15.0; Stata Corporation, College Station, TX, USA).

## Results

### Study and patient characteristics

**Fig. 1:**
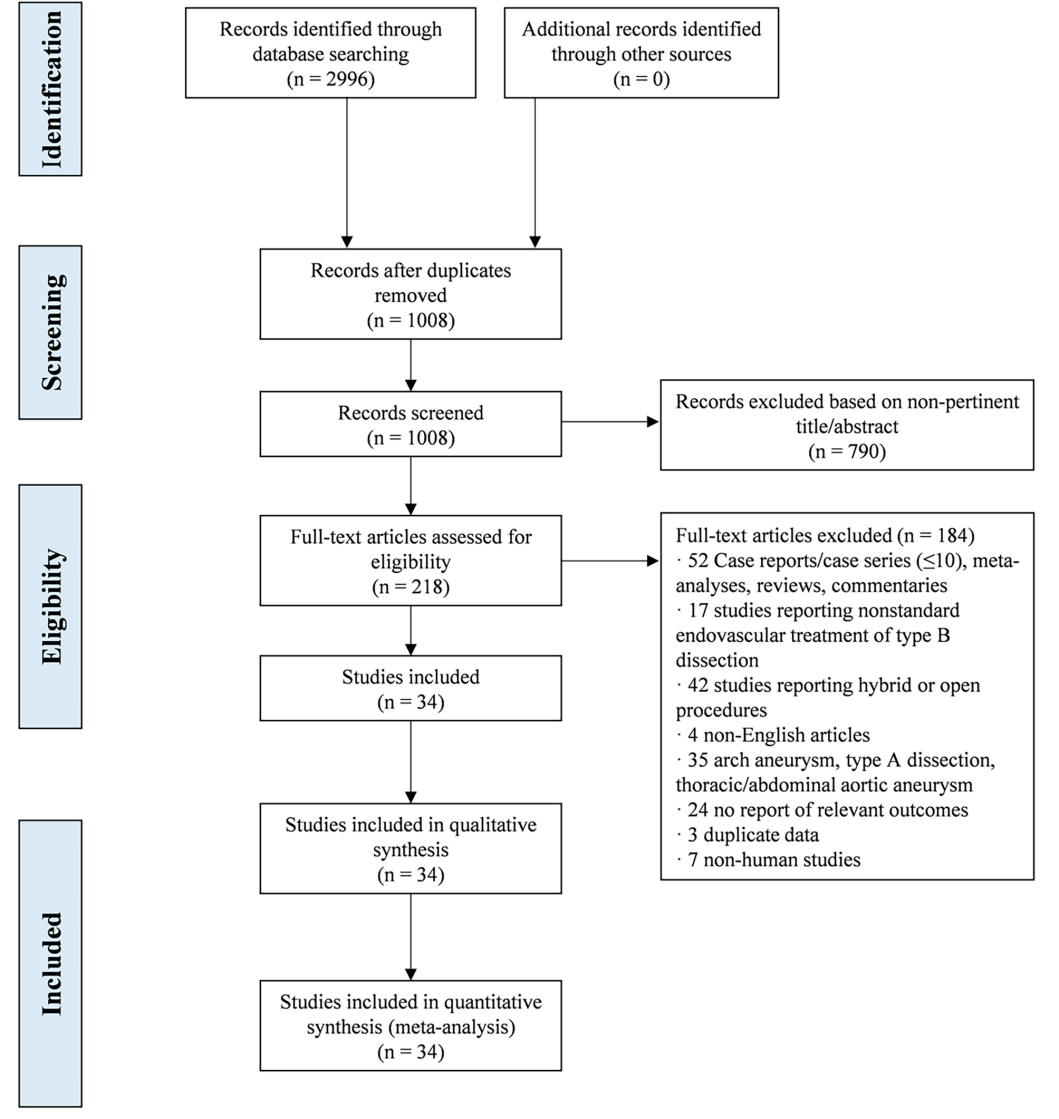
Preferred reporting items for systematic reviews and meta-analyses (PRISMA) flowchart of our analysis

In this analysis, the PRISMA statement flowchart explains the process of the evidence screening, inclusion and exclusion reasons (Fig. 1). A total of 1008 studies were screened, of which 34 studies with 2749 patients met the inclusion criteria. The baseline characteristics of the individual studies are summarized in Table 1. None of the studies was a randomized trial, and none had involved mutually overlapping populations. A full overview of all the extracted data and full references is summarized in Supplementary table S1.

**Table 1:** Summary of the included studies

Study	Study type	Study period	Location	Total patients	Total SCI, No. (%)	Downs and Black score	
Afifi 2015	Retrospective, single center	2001–2014	United States	37	2 (5.4)	6	
Andacheh 2012	Prospective, single center	2002–2010	United States	72	1 (1.4)	8	
Andersen 2014	Retrospective, single center	2005–2013	United States	44	0	8	
Cambria 2015	Prospective, multicenter	2010–2012	United States	50	4 (8.0)	10	
Chaikof 2009	Retrospective, single center	1998–2007	United States	44	1 (2.2)	6	
Chou 2015	Retrospective, single center	2003–2009	Taiwan	119	1 (0.8)	8	
Clough 2014	Retrospective, single center	2000–2014	United Kingdom	116	7 (6.0)	10	
Conway 2018	Retrospective, single center	2010–2015	United States	125	7 (5.6)	11	
Criado 2002	Retrospective, single center	1999–2002	United States	16	0	5	
Eleshra 2020	Retrospective, single center	2010–2017	Germany	64	2 (3.1)	5	
Hiraoka 2018	Retrospective, single center	2008–2014	Japan	64	3 (4.7)	9	
Jia 2013	Prospective, multicenter	2007–2010	China	208	2 (1.0)	11	
Katayama 2015	Retrospective, single center	1997–2011	Japan	144	2 (1.4)	12	
Lopez 2020	Retrospective, multicenter	2012–2016	Spain	90	6 (6.7)	8	
Lou 2023	Retrospective, single center	2012–2020	United States	50	2 (4.0)	8	
Mastroroberto 2010	Retrospective, single center	2001–2008	Italy	13	1 (7.7)	9	
Morales 2007	Retrospective, single center	1997–2006	United Kingdom	52	1 (1.9)	11	
Nozdrzykowski 2013	Retrospective, single center	2000–2010	Germany	32	3 (9.4)	8	
Oberhuber 2011	Retrospective, single center	1999–2011	Germany	19	1 (5.3)	6	
Preventza 2009	Prospective, single center	2000–2008	United States	109	4 (3.7)	9	
Qu 2008	Retrospective, single center	2005–2007	China	41	0	11	
Ricco 2006	Retrospective, multicenter	1999–2001	France	33	3 (9.0)	10	
Sandroussi 2007	Retrospective, single center	1995–2005	United Kingdom	23	0	7	
Scali 2013	Retrospective, single center	2004–2011	United States	80	8 (10.0)	11	
Sobocinski 2020	Retrospective, multicenter	2005–2015	United States	41	2 (4.9)	9	
Spinelli 2023	Prospective, multicenter	2010–2016	Italy	102	3 (2.9)	10	
Stelzmueller 2019	Retrospective, single center	2001–2016	Austria	55	3 (5.5)	12	
Ullery 2011	Retrospective, single center	2002–2010	United States	80	4 (5.0)	8	
Wamala 2022	Retrospective, single center	2009–2019	Germany	65	3 (4.6)	9	
Wang 2019	Retrospective, multicenter	2013–2016	United States	397	13 (3.3)	8	
Wilkinson 2013	Retrospective, single center	1995–2012	United States	49	3 (6.1)	5	
Zeeshan 2010	Retrospective, single center	2002–2010	United States	45	6 (13.3)	5	
Zhang 2018	Retrospective, multicenter	2013–2018	China	106	1 (0.9)	11	
Zipfel 2013	Prospective, single center	2000–2010	Germany	164	2 (1.2)	12	

SCI, spinal cord ischemia

### Study quality

The Downs and Black score were used to assess the quality of all 34 studies. The average score was 8.71 (range, 5–12).

### Permanent SCI rates after TEVAR with vs. without prophylactic CSFD

**Fig. 2:**
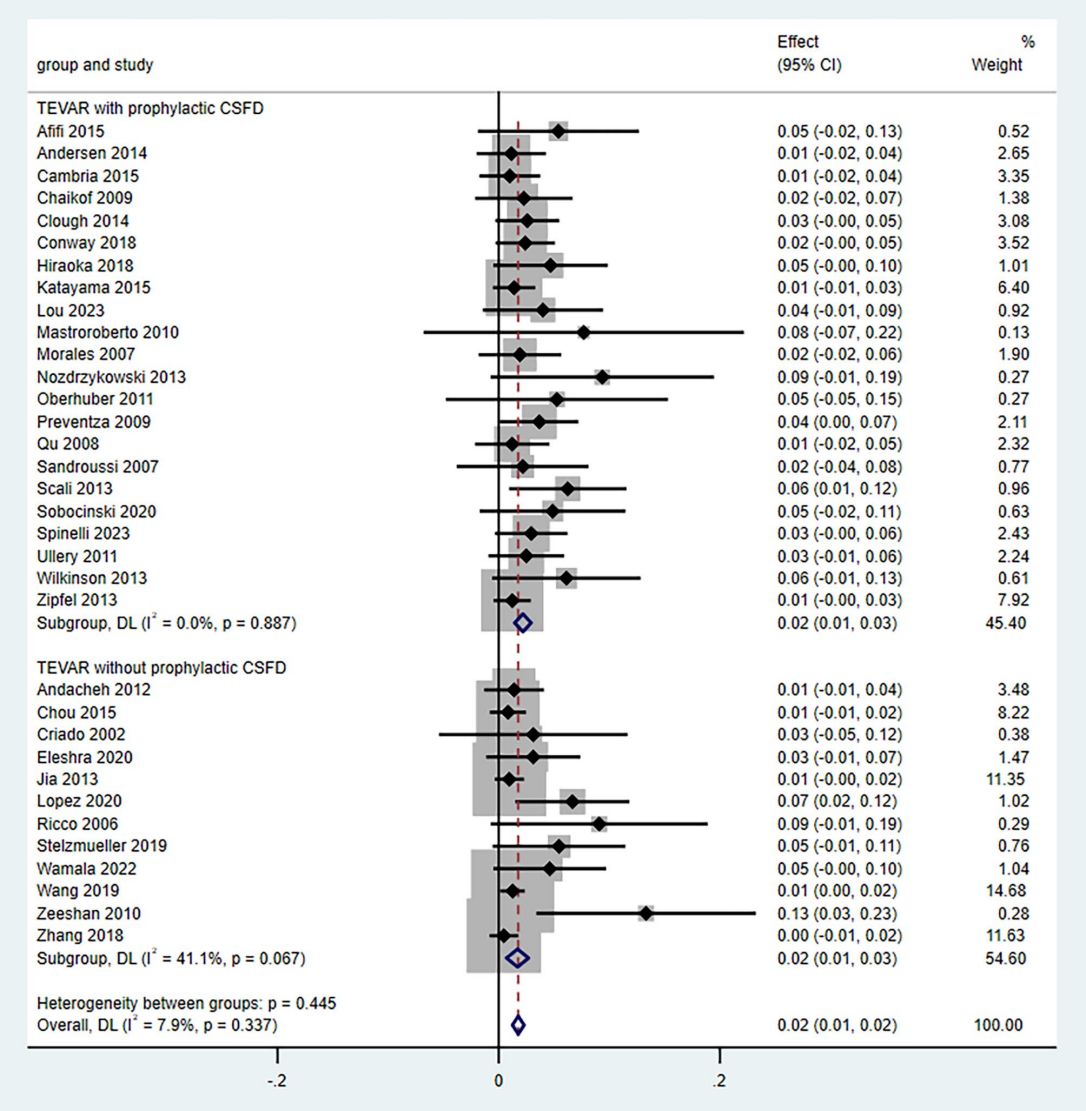
Forest plot using subgroup analysis to compare spinal cord ischemia (SCI) rates with vs. without prophylactic cerebrospinal fluid drainage (CSFD). CI: confidence interval; CSFD, cerebrospinal fluid drainage; TEVAR, thoracic endovascular aortic repair

The pooled ER for permanent SCI after TEVAR were 2.0% (95% CI, 1.0–2.0). The heterogeneity was not considered statistically significant (*P* = 0.337; *I*^*2*^ = 7.9%). No statistically significant differences were found in the estimates of the effects in the sensitivity analyses (Supplementary Fig. 1). Visual asymmetry was found in the funnel plot (Supplementary Fig. 2), and statistically significant *P* values were obtained using the Egger test (coefficient, 1.54; 95% CI, 1.20–1.87; *P* < 0.001), suggesting the existence of a publication bias (Supplementary Fig. 3). The subgroup analysis on the comparison of the permanent SCI rates with and without prophylactic CSFD is presented in Fig. 2. The subgroup statistical analysis showed that the permanent SCI rate with prophylactic CSFD was identical to that without prophylactic CSFD (2.0%; 95% CI, 1.0–3.0; *P* = 0.445).

### Permanent SCI rates after TEVAR with routine vs. selective prophylactic CSFD

**Fig. 3:**
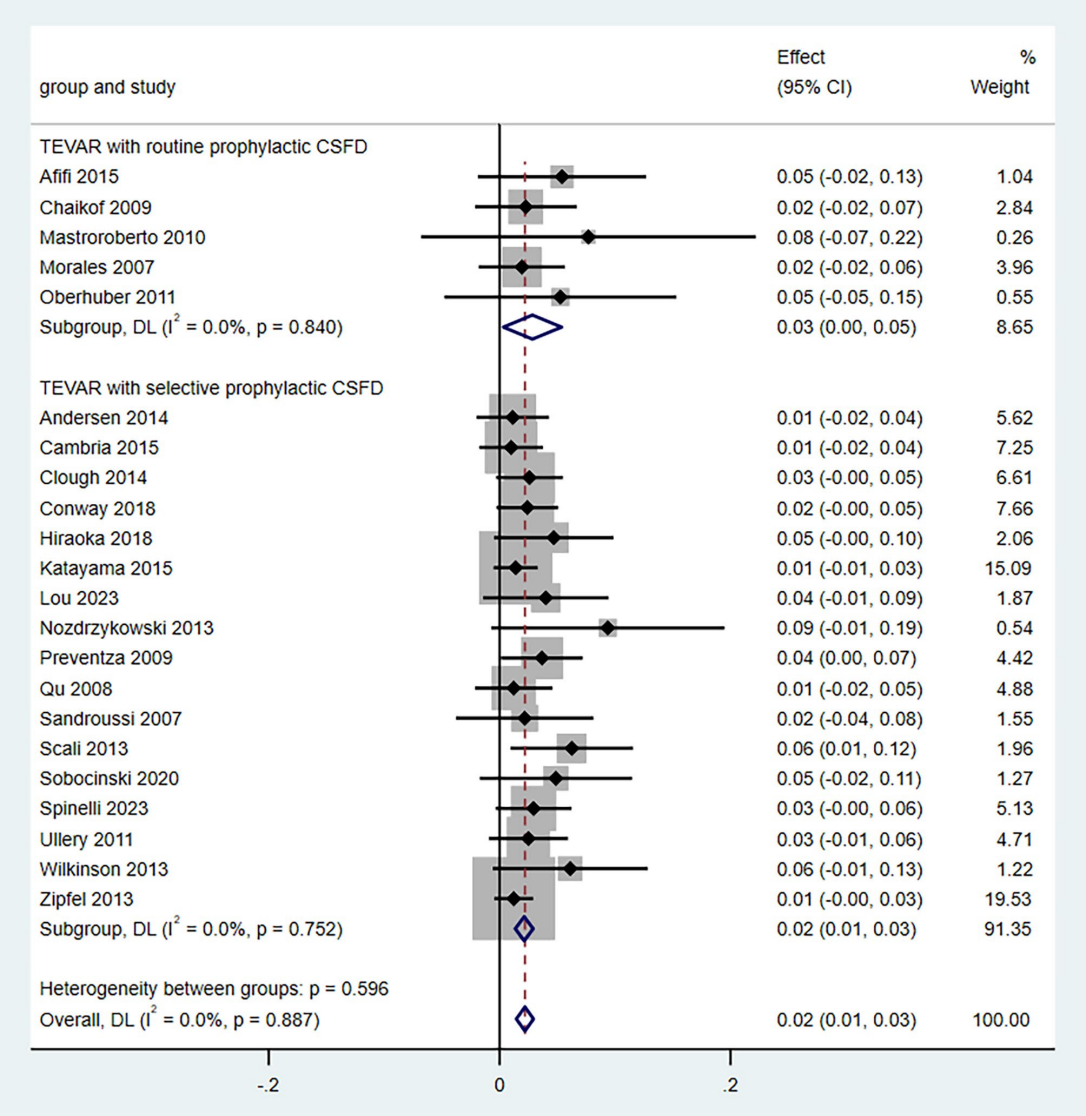
Forest plot using subgroup analysis to compare permanent spinal cord ischemia (SCI) rates between routine and selective prophylactic cerebrospinal fluid drainage (CSFD). CI: confidence interval; CSFD, cerebrospinal fluid drainage; TEVAR, thoracic endovascular aortic repair

A total of 22 studies with 1479 patients had reported the permanent SCI rates after TEVAR with prophylactic CSFD, yielding a pooled ER of 2.0% (95% CI, 1.0–3.0). The heterogeneity was considered not statistically significant (*P* = 0.887; *I*^*2*^ = 0.0%). No statistically significant differences were found in the estimates of the effects in the sensitivity analyses (Supplementary Fig. 1). Visual asymmetry was found in the funnel plot (Supplementary Fig. 2), and statistically significant *P* values were obtained using the Egger test (coefficient, 1.41; 95% CI, 0.95–1.88; *P* < 0.001), suggesting the existence of a publication bias (Supplementary Fig. 3). The subgroup analysis for the comparison of permanent SCI rates between routine and selective prophylactic CSFD is presented in Fig. 3. The results from the subgroup analysis showed that the permanent SCI rates with routine prophylactic CSFD (3.0%; 95% CI, 0.0–5.0) was not significantly different from that with selective prophylactic CSFD (2.0%; 95% CI, 1.0–3.0) for patients undergoing TEVAR for TBAD (*P* = 0.596).

### Temporary SCI rates after TEVAR

**Fig. 4:**
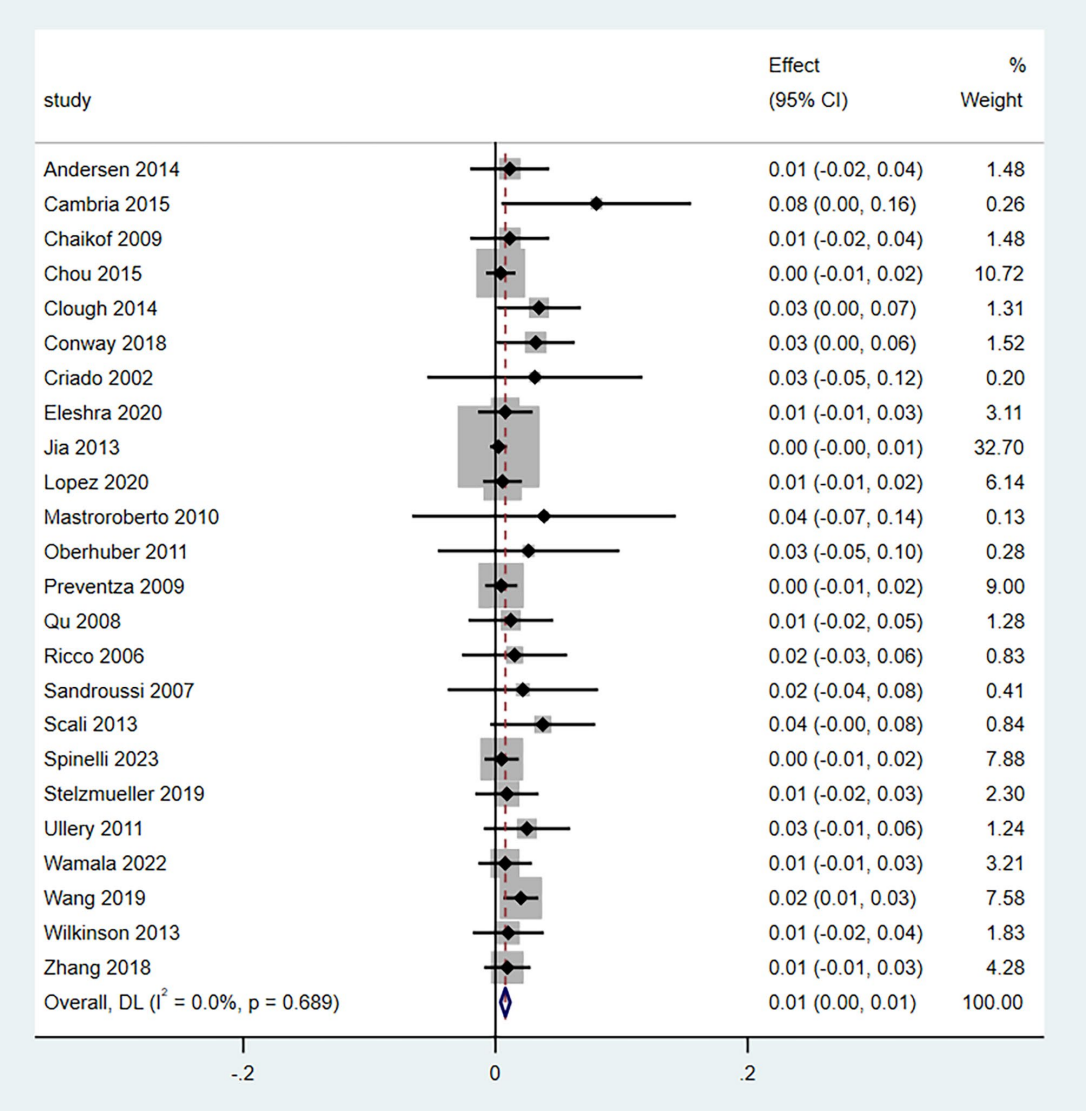
Forest plot for pooled rate of temporary spinal cord injury. CI: confidence interval; CSFD, cerebrospinal fluid drainage; TEVAR, thoracic endovascular aortic repair

A total of 24 studies with 2048 patients had reported the temporary SCI rates after TEVAR, yielding a pooled ER of 1.0% (95% CI, 0.00–1.0%) (Fig. 4). The heterogeneity was considered not statistically significant (*P* = 0.689; *I*^*2*^ = 0.0%). No statistically significant differences were found in the estimates of the effects in the sensitivity analyses (Supplementary Fig. 1). Visual asymmetry was found in the funnel plot (Supplementary Fig. 2), and statistically significant *P* values were obtained using the Egger test (coefficient, 1.13; 95% CI, 0.67–1.60; *P* < 0.001), suggesting the existence of a publication bias (Supplementary Fig. 3).

### 30-day or in-hospital mortality

**Fig. 5:**
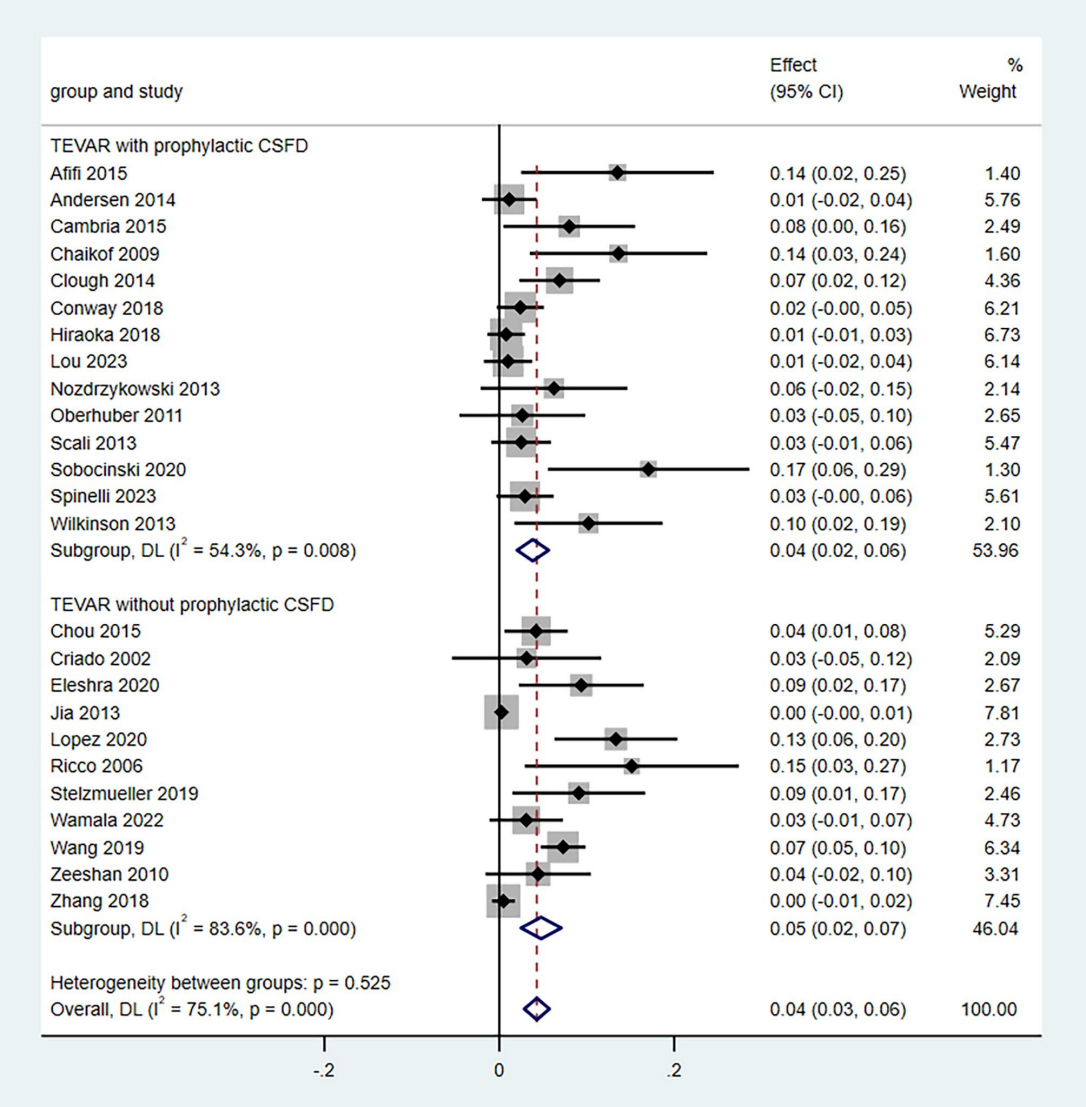
Forest plot using subgroup analysis to compare 30-day or in-hospital mortality with vs. without prophylactic cerebrospinal fluid drainage (CSFD). CI: confidence interval; CSFD, cerebrospinal fluid drainage; TEVAR, thoracic endovascular aortic repair

There were 25 studies with 2051 patients included in the analysis for 30-day or in-hospital mortality. The pooled rate for 30-day or in-hospital mortality was 4.0% (95% CI, 3.0–6.0) (Fig. 5). The heterogeneity was considered statistically significant (*P* = 0.000; *I*^*2*^ = 75.1%). No statistically significant differences were found in the estimates of the effects in the sensitivity analyses (Supplementary Fig. 1). Visual asymmetry was found in the funnel plot (Supplementary Fig. 2), and statistically significant *P* values were obtained using the Egger test (coefficient, 2.29; 95% CI, 1.65–2.93; *P* < 0.001), suggesting the existence of a publication bias (Supplementary Fig. 3). The subgroup analysis on the comparison of the 30-day or in-hospital mortality with and without prophylactic CSFD is presented in Fig. 5. The subgroup statistical analysis showed that the 30-day or in-hospital mortality was not significantly different (*P* = 0.525) in patients with prophylactic CSFD (4.0, 95% CI 2.0–6.0) or without prophylactic CSFD (5.0, 95% CI 2.0–7.0).

### Drain-related complications

The complication rates of CSFD were either inadequately reported or not even reported in a relevant portion of the studies. Accordingly, the present analysis regarding the drain-related complications was not performed due to lack of data.

## Discussion

This systematic review of 34 studies including 2749 patients can be summarized as follows: (1) The permanent SCI rate with prophylactic CSFD was identical to that without prophylactic CSFD (2.0%; 95% CI, 1.0–3.0; *P* = 0.445); (2) No statistically significant difference was found between the rates of permanent SCI with routine vs. selective prophylactic CSFD (*P* = 0.596); (3) The pooled rate of temporary SCI was 1.0% (95% CI, 0.00–1.0%); (4) the 30-day or in-hospital mortality was not significantly different (*P* = 0.525) in patients with prophylactic CSFD (4.0, 95% CI 2.0–6.0) or without prophylactic CSFD (5.0, 95% CI 2.0–7.0).

The reported incidence of permanent SCI remains variable across literature, with some reports showing TEVAR having higher postoperative SCI rates [24, 25] and others showing the opposite [26, 27]. In 2022, Zhang and colleagues [28] meta-analyzed the incidence of paraplegia in 14 studies on patients receiving CSFD and found the pooled odds ratio for development of paraplegia to be 1.80 (95% CI, 0.88–2.72) for CSFD use and 3.20 (95% CI, 0.81–7.20) for no CSFD use. In our analysis, we found the overall pooled rate of permanent SCI to be 2.0% (95% CI, 1.0–2.0). The subgroup statistical analysis showed that the permanent SCI rate after TEVAR with prophylactic CSFD was identical to that without prophylactic CSFD, suggesting that prophylactic CSFD might not be necessary for patients undergoing TEVAR for TBAD. Therefore, it is possible that CSFD use was not explicitly reported in some of the studies or selectively not used only in low-risk patients. What’s more, our sample size was much larger and included patients from different countries; therefore, our results could be generalized on a greater scale.

Permanent SCI after TEVAR exerts a devastating impact on patient’s quality of life and life expectancy. A retrospective review of 607 TEVAR patients revealed mean postoperative survival of 37.2 ± 4.5 months in patients who developed SCI, compared with 71.6 ± 3.9 months (*P* < 0.0006) for those who did not develop SCI. Patients with SCI who manifested functional improvement showed much-improved survival of 53.9 ± 5.9 months compared with 9.6 ± 3.6 months for those with a permanent neurological deficit (*P* < 0.0001) [29]. The present analysis showed that the 30-day or in-hospital mortality was not significantly different in patients with prophylactic CSFD or without prophylactic CSFD (*P* = 0.525), suggesting that prophylactic CSFD might not be associated with a reduction in 30-day or in-hospital mortality. Therefore, prophylactic CSFD was not related to favorable outcome regarding 30-day or in-hospital mortality after endovascular repair of TBAD.

The risk of SCI following TEVAR varies and depends primarily on the extent of coverage of the segmental arteries and the vigor of the paraspinal collateral network. Several risk factors predispose TEVAR patients to SCI [30, 31]. These include severe calcification or extensive coverage of the descending thoracic aorta (≥ 20 cm long), coverage of the left subclavian artery without revascularization, coverage of the celiac artery, or occlusion of the hypogastric plexus. Because the prognosis after the development of permanent SCI is usually dismal and permanently affects patients’ quality of life when it occurs, the risks of SCI should always be assessed preoperatively. Prevention measures should be considered for high-risk patients.

SCI protection protocols often benefit from a multimodal approach in preventing spinal cord injury, including staging with temporary aneurysm sac perfusion (TASP), permissive hypertension, and CSFD (prophylactic or emergency) [32, 33]. Additionally, other pre-operative staging techniques such as minimally invasive segmental artery coil embolization (MISACE) may be related to improved spinal cord collateralization leading to reduced SCI rates [34]. Although CSFD is considered the most effective prevention and treatment of SCI, the risk of CSFD-related complications is not negligible and should be carefully weighed. An ongoing change of concept from prophylactic CSFD to emergency CSFD in case of onset of SCI has been described in the literature lately. Moher et al [35] reported that one third of SCI were caused by prophylactic CSFD placement. Additionally, Marcondes et al. described low mortality rates and low rates of permanent paraplegia (2%) without the routine use of prophylactic CSFD preoperatively [36]. Therefore, prophylactic CSFD should be selectively, but not routinely, used for patients with TBAD with the stated risk factors.

### Limitations

The present systematic review had some limitations. First, the definitions of high risk could have varied among the included publications, which could have resulted in inconsistencies for the choice of routine vs. selective prophylactic CSFD. Second, due to the lack of patient-level data to assess the exact risk factors for each patient, the systematic review was unable to exclude the effects of other prophylactic measures preventing SCI. Third, the definition of SCI and mentioning of permanent vs. temporary SCI was different among the studies, which in turn may lead to bias and the impossibility of differentiating between the types of SCI. Fourth, scarce data on the extent of TBAD, the length of aortic coverage by TEVAR, and CSFD-related complications were provided in the eligible studies. Fifth, the 34 included studies received a Downs and Black score of ≤ 12, qualifying as poor. Sixth, all funnel plots were asymmetric, together with statistically significant *P* values obtained using the Egger test, suggesting the existence of a possible publication bias in the outcome measures. Finally, most included reports had been retrospective studies and lacked data for the assessment of bias owing to confounding.

## Conclusions

Prophylactic CSFD was not associated with a lower rate of permanent SCI and 30-day or in-hospital mortality after TEVAR for TBAD. Due to the low quality of evidence, no clear recommendation on the use of prophylactic CSFD can be made.

### Electronic supplementary material

Below is the link to the electronic supplementary material.

Supplementary Material 1

**Supplementary Fig. 1.** Sensitivity analyses for all outcome measures. CSFD, Cerebrospinal fluid drainage; SCI, spinal cord ischemia; TEVAR, thoracic endovascular aortic repair

**Supplementary Fig. 2.** Funnel plots for all outcome measures. CSFD, Cerebrospinal fluid drainage; SCI, spinal cord ischemia; TEVAR, thoracic endovascular aortic repair

**Supplementary Fig. 3.** Egger test for all outcome measures. CSFD, Cerebrospinal fluid drainage; SCI, spinal cord ischemia; TEVAR, thoracic endovascular aortic repair

Supplementary Material 5

## Data Availability

All data generated or analyzed during this study are included in this published article and its supplementary information files.

## Abbreviations

CI: Confidence interval
CSF: Cerebrospinal fluid
CSFD: Cerebrospinal fluid drainage
ER: Event rate
SCI: Spinal cord ischemia
TEVAR: Thoracic endovascular aortic repair
TBAD: Type B aortic dissection
