# Supplementary material for: Systematic review of the effect of cerebrospinal fluid drainage on outcomes after endovascular type B aortic dissection repair
Source: J Cardiothorac Surg. 2024 Mar 12;19:116. doi: 10.1186/s13019-024-02603-3 (PMC10935911; doi:10.1186/s13019-024-02603-3)
Supplement: Supplementary file 1 — Supplementary Material 1 [file 13019_2024_2603_MOESM1_ESM.docx]

**Supplementary Appendix**

**Search strategies:**

**Source Searched – PubMed**

**Searched on May 7, 2023**

**Limits- None**

**Results: 940**

**Search Strategy** – ("Aortic Dissection"[MeSH Terms] OR ("Aortic Dissection"[MeSH Terms] OR ("aortic"[All Fields] AND "dissection"[All Fields]) OR "Aortic Dissection"[All Fields] OR ("aortic"[All Fields] AND "dissections"[All Fields]) OR "aortic dissections"[All Fields] OR ("Aortic Dissection"[MeSH Terms] OR ("aortic"[All Fields] AND "dissection"[All Fields]) OR "Aortic Dissection"[All Fields] OR ("dissection"[All Fields] AND "aortic"[All Fields]) OR "dissection aortic"[All Fields]) OR ("Aortic Dissection"[MeSH Terms] OR ("aortic"[All Fields] AND "dissection"[All Fields]) OR "Aortic Dissection"[All Fields] OR ("aortic"[All Fields] AND "dissecting"[All Fields] AND "aneurysm"[All Fields]) OR "aortic dissecting aneurysm"[All Fields]) OR ("Aortic Dissection"[MeSH Terms] OR ("aortic"[All Fields] AND "dissection"[All Fields]) OR "Aortic Dissection"[All Fields] OR ("aneurysm"[All Fields] AND "aortic"[All Fields] AND "dissecting"[All Fields])) OR ("Aortic Dissection"[MeSH Terms] OR ("aortic"[All Fields] AND "dissection"[All Fields]) OR "Aortic Dissection"[All Fields] OR ("aortic"[All Fields] AND "dissecting"[All Fields] AND "aneurysms"[All Fields]) OR "aortic dissecting aneurysms"[All Fields]) OR ("Aortic Dissection"[MeSH Terms] OR ("aortic"[All Fields] AND "dissection"[All Fields]) OR "Aortic Dissection"[All Fields] OR ("dissecting"[All Fields] AND "aneurysm"[All Fields] AND "aortic"[All Fields]) OR "dissecting aneurysm aortic"[All Fields]) OR ("Aortic Dissection"[MeSH Terms] OR ("aortic"[All Fields] AND "dissection"[All Fields]) OR "Aortic Dissection"[All Fields] OR ("dissecting"[All Fields] AND "aneurysm"[All Fields] AND "aorta"[All Fields]) OR "dissecting aneurysm aorta"[All Fields]) OR ("Aortic Dissection"[MeSH Terms] OR ("aortic"[All Fields] AND "dissection"[All Fields]) OR "Aortic Dissection"[All Fields] OR ("aneurysm"[All Fields] AND "aorta"[All Fields] AND "dissecting"[All Fields])) OR ("Aortic Dissection"[MeSH Terms] OR ("aortic"[All Fields] AND "dissection"[All Fields]) OR "Aortic Dissection"[All Fields] OR ("aorta"[All Fields] AND "dissecting"[All Fields] AND "aneurysm"[All Fields]) OR "aorta dissecting aneurysm"[All Fields]) OR ("Aortic Dissection"[MeSH Terms] OR ("aortic"[All Fields] AND "dissection"[All Fields]) OR "Aortic Dissection"[All Fields] OR ("dissecting"[All Fields] AND "aneurysm"[All Fields] AND "aortas"[All Fields])) OR ("dissection, blood vessel"[MeSH Terms] OR ("dissection"[All Fields] AND "blood"[All Fields] AND "vessel"[All Fields]) OR "blood vessel dissection"[All Fields] OR ("aneurysm"[All Fields] AND "dissecting"[All Fields]) OR "aneurysm dissecting"[All Fields]) OR ("Aortic Dissection"[MeSH Terms] OR ("aortic"[All Fields] AND "dissection"[All Fields]) OR "Aortic Dissection"[All Fields] OR ("dissecting"[All Fields] AND "aneurysms"[All Fields]) OR "dissecting aneurysms"[All Fields]) OR ("Aortic Dissection"[MeSH Terms] OR ("aortic"[All Fields] AND "dissection"[All Fields]) OR "Aortic Dissection"[All Fields] OR ("dissecting"[All Fields] AND "aneurysm"[All Fields]) OR "dissecting aneurysm"[All Fields]))) AND ("Stents"[All Fields] OR "stent graft"[All Fields] OR "endovascular"[All Fields]) AND ("Spinal Cord Ischemia"[MeSH Terms] OR ("Spinal Cord Ischemia"[MeSH Terms] OR ("spinal"[All Fields] AND "cord"[All Fields] AND "ischemia"[All Fields]) OR "Spinal Cord Ischemia"[All Fields] OR ("cord"[All Fields] AND "ischemia"[All Fields] AND "spinal"[All Fields]) OR "cord ischemia spinal"[All Fields] OR ("Spinal Cord Ischemia"[MeSH Terms] OR ("spinal"[All Fields] AND "cord"[All Fields] AND "ischemia"[All Fields]) OR "Spinal Cord Ischemia"[All Fields] OR ("cord"[All Fields] AND "ischemias"[All Fields] AND "spinal"[All Fields])) OR ("Spinal Cord Ischemia"[MeSH Terms] OR ("spinal"[All Fields] AND "cord"[All Fields] AND "ischemia"[All Fields]) OR "Spinal Cord Ischemia"[All Fields] OR ("ischemia"[All Fields] AND "spinal"[All Fields] AND "cord"[All Fields]) OR "ischemia spinal cord"[All Fields]) OR ("Spinal Cord Ischemia"[MeSH Terms] OR ("spinal"[All Fields] AND "cord"[All Fields] AND "ischemia"[All Fields]) OR "Spinal Cord Ischemia"[All Fields] OR ("ischemias"[All Fields] AND "spinal"[All Fields] AND "cord"[All Fields])) OR ("Spinal Cord Ischemia"[MeSH Terms] OR ("spinal"[All Fields] AND "cord"[All Fields] AND "ischemia"[All Fields]) OR "Spinal Cord Ischemia"[All Fields] OR ("spinal"[All Fields] AND "cord"[All Fields] AND "ischemias"[All Fields]) OR "spinal cord ischemias"[All Fields]) OR ("ischaemic myelopathy"[All Fields] OR "Spinal Cord Ischemia"[MeSH Terms] OR ("spinal"[All Fields] AND "cord"[All Fields] AND "ischemia"[All Fields]) OR "Spinal Cord Ischemia"[All Fields] OR ("ischemic"[All Fields] AND "myelopathy"[All Fields]) OR "ischemic myelopathy"[All Fields]) OR ("Spinal Cord Ischemia"[MeSH Terms] OR ("spinal"[All Fields] AND "cord"[All Fields] AND "ischemia"[All Fields]) OR "Spinal Cord Ischemia"[All Fields] OR ("ischemic"[All Fields] AND "myelopathies"[All Fields]) OR "ischemic myelopathies"[All Fields]) OR ("Spinal Cord Ischemia"[MeSH Terms] OR ("spinal"[All Fields] AND "cord"[All Fields] AND "ischemia"[All Fields]) OR "Spinal Cord Ischemia"[All Fields] OR ("myelopathies"[All Fields] AND "ischemic"[All Fields])) OR ("Spinal Cord Ischemia"[MeSH Terms] OR ("spinal"[All Fields] AND "cord"[All Fields] AND "ischemia"[All Fields]) OR "Spinal Cord Ischemia"[All Fields] OR ("myelopathy"[All Fields] AND "ischemic"[All Fields])) OR ("experimental spinal cord ischaemia"[All Fields] OR "Spinal Cord Ischemia"[MeSH Terms] OR ("spinal"[All Fields] AND "cord"[All Fields] AND "ischemia"[All Fields]) OR "Spinal Cord Ischemia"[All Fields] OR ("experimental"[All Fields] AND "spinal"[All Fields] AND "cord"[All Fields] AND "ischemia"[All Fields]) OR "experimental spinal cord ischemia"[All Fields]) OR ("Spinal Cord Ischemia"[MeSH Terms] OR ("spinal"[All Fields] AND "cord"[All Fields] AND "ischemia"[All Fields]) OR "Spinal Cord Ischemia"[All Fields] OR ("spinal"[All Fields] AND "cord"[All Fields] AND "ischemia"[All Fields] AND "experimental"[All Fields]) OR "spinal cord ischemia experimental"[All Fields])) OR ("Paraplegia"[MeSH Terms] OR ("Paraplegia"[MeSH Terms] OR "Paraplegia"[All Fields] OR "paraplegias"[All Fields] OR ("Paraplegia"[MeSH Terms] OR "Paraplegia"[All Fields] OR ("paralysis"[All Fields] AND "legs"[All Fields])) OR ("Paraplegia"[MeSH Terms] OR "Paraplegia"[All Fields] OR ("paralysis"[All Fields] AND "lower"[All Fields] AND "limbs"[All Fields]) OR "paralysis lower limbs"[All Fields]) OR ("Paraplegia"[MeSH Terms] OR "Paraplegia"[All Fields] OR ("paralysis"[All Fields] AND "lower"[All Fields] AND "extremities"[All Fields])) OR ("Paraplegia"[MeSH Terms] OR "Paraplegia"[All Fields] OR ("Paraplegia"[All Fields] AND "spinal"[All Fields]) OR "paraplegia spinal"[All Fields]) OR ("Paraplegia"[MeSH Terms] OR "Paraplegia"[All Fields] OR ("paraplegias"[All Fields] AND "spinal"[All Fields]) OR "paraplegias spinal"[All Fields]) OR ("Paraplegia"[MeSH Terms] OR "Paraplegia"[All Fields] OR ("spinal"[All Fields] AND "Paraplegia"[All Fields]) OR "spinal paraplegia"[All Fields]) OR ("Paraplegia"[MeSH Terms] OR "Paraplegia"[All Fields] OR ("spinal"[All Fields] AND "paraplegias"[All Fields]) OR "spinal paraplegias"[All Fields]) OR ("Paraplegia"[MeSH Terms] OR "Paraplegia"[All Fields] OR ("Paraplegia"[All Fields] AND "flaccid"[All Fields]) OR "paraplegia flaccid"[All Fields]) OR ("Paraplegia"[MeSH Terms] OR "Paraplegia"[All Fields] OR ("flaccid"[All Fields] AND "Paraplegia"[All Fields]) OR "flaccid paraplegia"[All Fields]) OR ("Paraplegia"[MeSH Terms] OR "Paraplegia"[All Fields] OR ("flaccid"[All Fields] AND "paraplegias"[All Fields])) OR ("Paraplegia"[MeSH Terms] OR "Paraplegia"[All Fields] OR ("paraplegias"[All Fields] AND "flaccid"[All Fields])) OR ("Paraplegia"[MeSH Terms] OR "Paraplegia"[All Fields] OR ("Paraplegia"[All Fields] AND "spastic"[All Fields]) OR "paraplegia spastic"[All Fields]) OR ("Paraplegia"[MeSH Terms] OR "Paraplegia"[All Fields] OR ("paraplegias"[All Fields] AND "spastic"[All Fields])) OR ("Paraplegia"[MeSH Terms] OR "Paraplegia"[All Fields] OR ("spastic"[All Fields] AND "paraplegias"[All Fields]) OR "spastic paraplegias"[All Fields]) OR ("Paraplegia"[MeSH Terms] OR "Paraplegia"[All Fields] OR ("spastic"[All Fields] AND "Paraplegia"[All Fields]) OR "spastic paraplegia"[All Fields]) OR ("Paraplegia"[MeSH Terms] OR "Paraplegia"[All Fields] OR ("Paraplegia"[All Fields] AND "ataxic"[All Fields]) OR "paraplegia ataxic"[All Fields]) OR ("Paraplegia"[MeSH Terms] OR "Paraplegia"[All Fields] OR ("ataxic"[All Fields] AND "Paraplegia"[All Fields]) OR "ataxic paraplegia"[All Fields]) OR ("Paraplegia"[MeSH Terms] OR "Paraplegia"[All Fields] OR ("ataxic"[All Fields] AND "paraplegias"[All Fields])) OR ("Paraplegia"[MeSH Terms] OR "Paraplegia"[All Fields] OR ("paraplegias"[All Fields] AND "ataxic"[All Fields])) OR ("Paraplegia"[MeSH Terms] OR "Paraplegia"[All Fields] OR ("Paraplegia"[All Fields] AND "cerebral"[All Fields]) OR "paraplegia cerebral"[All Fields]) OR ("Paraplegia"[MeSH Terms] OR "Paraplegia"[All Fields] OR ("cerebral"[All Fields] AND "Paraplegia"[All Fields]) OR "cerebral paraplegia"[All Fields]) OR ("Paraplegia"[MeSH Terms] OR "Paraplegia"[All Fields] OR ("cerebral"[All Fields] AND "paraplegias"[All Fields])) OR ("Paraplegia"[MeSH Terms] OR "Paraplegia"[All Fields] OR ("paraplegias"[All Fields] AND "cerebral"[All Fields])))) OR ("Paraparesis"[MeSH Terms] OR ("Paraparesis"[MeSH Terms] OR "Paraparesis"[All Fields] OR "parapareses"[All Fields] OR ("Paraparesis"[MeSH Terms] OR "Paraparesis"[All Fields] OR ("Paraparesis"[All Fields] AND "spinal"[All Fields]) OR "paraparesis spinal"[All Fields]) OR ("Paraparesis"[MeSH Terms] OR "Paraparesis"[All Fields] OR ("spinal"[All Fields] AND "Paraparesis"[All Fields]) OR "spinal paraparesis"[All Fields]) OR ("Paraparesis"[MeSH Terms] OR "Paraparesis"[All Fields] OR ("Paraparesis"[All Fields] AND "hypotonic"[All Fields])) OR ("Paraparesis"[MeSH Terms] OR "Paraparesis"[All Fields] OR ("hypotonic"[All Fields] AND "Paraparesis"[All Fields]) OR "hypotonic paraparesis"[All Fields]) OR ("Paraparesis"[MeSH Terms] OR "Paraparesis"[All Fields] OR ("Paraparesis"[All Fields] AND "cerebral"[All Fields]) OR "paraparesis cerebral"[All Fields]) OR ("Paraparesis"[MeSH Terms] OR "Paraparesis"[All Fields] OR ("cerebral"[All Fields] AND "Paraparesis"[All Fields])) OR ("Paraparesis"[MeSH Terms] OR "Paraparesis"[All Fields] OR ("Paraparesis"[All Fields] AND "chronic"[All Fields] AND "progressive"[All Fields])) OR ("Paraparesis"[MeSH Terms] OR "Paraparesis"[All Fields] OR ("chronic"[All Fields] AND "progressive"[All Fields] AND "Paraparesis"[All Fields]) OR "chronic progressive paraparesis"[All Fields]) OR ("Paraparesis"[MeSH Terms] OR "Paraparesis"[All Fields] OR ("progressive"[All Fields] AND "Paraparesis"[All Fields] AND "chronic"[All Fields])))))

**Source Searched – Embase**

**Searched on May 7, 2023**

**Limits- None**

**Results: 1098**

**Search Strategy** – ('aortic dissection'/exp OR 'aortic dissections':ab,ti OR 'dissection, aortic':ab,ti OR 'aortic dissecting aneurysm':ab,ti OR 'aneurysm, aortic dissecting':ab,ti OR 'aortic dissecting aneurysms':ab,ti OR 'dissecting aneurysm, aortic':ab,ti OR 'dissecting aneurysm aorta':ab,ti OR 'aneurysm aorta, dissecting':ab,ti OR 'aorta, dissecting aneurysm':ab,ti OR 'dissecting aneurysm aortas':ab,ti OR 'aneurysm, dissecting':ab,ti OR 'dissecting aneurysms':ab,ti OR 'dissecting aneurysm':ab,ti) AND ('paraplegia'/exp OR 'paraplegias':ab,ti OR 'paralysis, legs':ab,ti OR 'paralysis, lower limbs':ab,ti OR 'paralysis, lower extremities':ab,ti OR 'paraplegia, spinal':ab,ti OR 'paraplegias, spinal':ab,ti OR 'spinal paraplegia':ab,ti OR 'spinal paraplegias':ab,ti OR 'paraplegia, flaccid':ab,ti OR 'flaccid paraplegia':ab,ti OR 'flaccid paraplegias':ab,ti OR 'paraplegias, flaccid':ab,ti OR 'paraplegia, spastic':ab,ti OR 'paraplegias, spastic':ab,ti OR 'spastic paraplegias':ab,ti OR 'spastic paraplegia':ab,ti OR 'paraplegia, ataxic':ab,ti OR 'ataxic paraplegia':ab,ti OR 'ataxic paraplegias':ab,ti OR 'paraplegias, ataxic':ab,ti OR 'paraplegia, cerebral':ab,ti OR 'cerebral paraplegia':ab,ti OR 'cerebral paraplegias':ab,ti OR 'paraplegias, cerebral':ab,ti OR 'paraparesis':ab,ti OR 'parapareses':ab,ti OR 'paraparesis, spinal':ab,ti OR 'spinal paraparesis':ab,ti OR 'paraparesis, hypotonic':ab,ti OR 'hypotonic paraparesis':ab,ti OR 'paraparesis, cerebral':ab,ti OR 'cerebral paraparesis':ab,ti OR 'paraparesis, chronic progressive':ab,ti OR 'chronic progressive paraparesis':ab,ti OR 'progressive paraparesis, chronic':ab,ti OR 'spinal cord ischemia'/exp OR 'cord ischemia, spinal':ab,ti OR 'cord ischemias, spinal':ab,ti OR 'ischemia, spinal cord':ab,ti OR 'ischemias, spinal cord':ab,ti OR 'spinal cord ischemias':ab,ti OR 'ischemic myelopathy':ab,ti OR 'ischemic myelopathies':ab,ti OR 'myelopathies, ischemic':ab,ti OR 'myelopathy, ischemic':ab,ti OR 'experimental spinal cord ischemia':ab,ti OR 'spinal cord ischemia, experimental':ab,ti) AND ('endovascular':ab,ti OR 'stents':ab,ti OR 'stent graft':ab,ti)

**Source Searched – Web of Science**

**Searched on May 7, 2023**

**Limits- None**

**Results: 901**

**Search Strategy:**

#1: Aortic Dissection (主题) OR Aortic Dissections (主题) OR Dissection, Aortic (主题) OR Aortic Dissecting Aneurysm (主题) OR Aneurysm, Aortic Dissecting (主题) OR Aortic Dissecting Aneurysms (主题) OR Dissecting Aneurysm, Aortic (主题) OR Dissecting Aneurysm Aorta (主题) OR Aneurysm Aorta, Dissecting (主题) OR Aorta, Dissecting Aneurysm (主题) OR Dissecting Aneurysm Aortas (主题) OR Aneurysm, Dissecting (主题) OR Dissecting Aneurysms (主题) OR Dissecting Aneurysm (主题) 检索结果: 27407

#2: Paraplegia (主题) AND Paraplegias (主题) OR Paralysis, Legs (主题) OR Paralysis, Lower Limbs (主题) OR Paralysis, Lower Extremities (主题) OR Paraplegia, Spinal (主题) OR Paraplegias, Spinal (主题) OR Spinal Paraplegia (主题) OR Spinal Paraplegias (主题) OR Paraplegia, Flaccid (主题) OR Flaccid Paraplegia (主题) OR Flaccid Paraplegias (主题) OR Paraplegias, Flaccid (主题) OR Paraplegia, Spastic (主题) OR Paraplegias, Spastic (主题) AND Spastic Paraplegias (主题) AND Spastic Paraplegia (主题) AND Paraplegia, Ataxic (主题) AND Ataxic Paraplegia (主题) AND Ataxic Paraplegias (主题) AND Paraplegias, Ataxic (主题) AND Paraplegia, Cerebral (主题) AND Cerebral Paraplegia (主题) AND Cerebral Paraplegias (主题) AND Paraplegias, Cerebral (主题) 检索结果: 17047

#3: Paraparesis (主题) OR Parapareses (主题) OR Paraparesis, Spinal (主题) OR Spinal Paraparesis (主题) OR Paraparesis, Hypotonic (主题) OR Hypotonic Paraparesis (主题) OR Paraparesis, Cerebral (主题) OR Cerebral Paraparesis (主题) OR Paraparesis, Chronic Progressive (主题) OR Chronic Progressive Paraparesis (主题) OR Progressive Paraparesis, Chronic (主题) 检索结果: 7188

#4: Spinal Cord Ischemia (主题) OR Cord Ischemia, Spinal (主题) OR Cord Ischemias, Spinal (主题) OR Ischemia, Spinal Cord (主题) OR Ischemias, Spinal Cord (主题) OR Spinal Cord Ischemias (主题) OR Ischemic Myelopathy (主题) OR Ischemic Myelopathies (主题) OR Myelopathies, Ischemic (主题) OR Myelopathy, Ischemic (主题) OR Experimental Spinal Cord Ischemia (标题) OR Spinal Cord Ischemia, Experimental (主题) 检索结果: 8526

#5: Stents (主题) OR stent graft (主题) OR endovascular (主题) 检索结果: 131801

#6: #2 OR #3 OR #4 检索结果: 30051

#7: #2 OR #3 OR #4 检索结果: 30051

#8: #7 AND #5 AND #1 检索结果: 901

**Source Searched – Cochrane Library**

**Searched on May 7, 2023**

**Limits- None**

**Results: 57**

**Search Strategy:**

#1 MeSH descriptor: [Aortic Dissection] explode all trees 145

#2 (Aortic Dissections OR Dissection, Aortic OR Aortic Dissecting Aneurysm OR Aneurysm, Aortic Dissecting OR Aortic Dissecting Aneurysms OR Dissecting Aneurysm, Aortic OR Dissecting Aneurysm Aorta OR Aneurysm Aorta, Dissecting OR Aorta, Dissecting Aneurysm OR Dissecting Aneurysm Aortas OR Aneurysm, Dissecting OR Dissecting Aneurysms OR Dissecting Aneurysm):ti,ab,kw (Word variations have been searched) 883

#3 #1 OR #2 883

#4 MeSH descriptor: [Paraplegia] explode all trees 290

#5 (Paraplegias OR Paralysis, Legs OR Paralysis, Lower Limbs OR Paralysis, Lower Extremities OR Paraplegia, Spinal OR Paraplegias, Spinal OR Spinal Paraplegia OR Spinal Paraplegias OR Paraplegia, Flaccid OR Flaccid Paraplegia OR Flaccid Paraplegias OR Paraplegias, Flaccid OR Paraplegia, Spastic OR Paraplegias, Spastic OR Spastic Paraplegias OR Spastic Paraplegia OR Paraplegia, Ataxic OR Ataxic Paraplegia OR Ataxic Paraplegias OR Paraplegias, Ataxic OR Paraplegia, Cerebral OR Cerebral Paraplegia OR Cerebral Paraplegias OR Paraplegias, Cerebral):ti,ab,kw (Word variations have been searched) 1076

#6 MeSH descriptor: [Paraparesis] explode all trees 23

#7 (Parapareses OR Paraparesis, Spinal OR Spinal Paraparesis OR Paraparesis, Hypotonic OR Hypotonic Paraparesis OR Paraparesis, Cerebral OR Cerebral Paraparesis OR Paraparesis, Chronic Progressive OR Chronic Progressive Paraparesis OR Progressive Paraparesis, Chronic):ti,ab,kw (Word variations have been searched) 70

#8 MeSH descriptor: [Spinal Cord Ischemia] explode all trees 20

#9 (Cord Ischemia, Spinal OR Cord Ischemias, Spinal OR Ischemia, Spinal Cord OR Ischemias, Spinal Cord OR Spinal Cord Ischemias OR Ischemic Myelopathy OR Ischemic Myelopathies OR Myelopathies, Ischemic OR Myelopathy, Ischemic OR Experimental Spinal Cord Ischemia OR Spinal Cord Ischemia, Experimental):ti,ab,kw (Word variations have been searched) 200

#10 #4 OR #5 1077

#11 (Stents OR stent graft OR endovascular):ti,ab,kw (Word variations have been searched) 21737

#12 #6 OR #7 84

#13 #8 OR #9 200

#14 #10 OR #12 OR #13 1292

#15 3# AND #14 AND #11 57
