# Supplementary figures and images for: Systematic review of the effect of cerebrospinal fluid drainage on outcomes after endovascular type B aortic dissection repair
Source: J Cardiothorac Surg. 2024 Mar 12;19:116. doi: 10.1186/s13019-024-02603-3 (PMC10935911; doi:10.1186/s13019-024-02603-3)

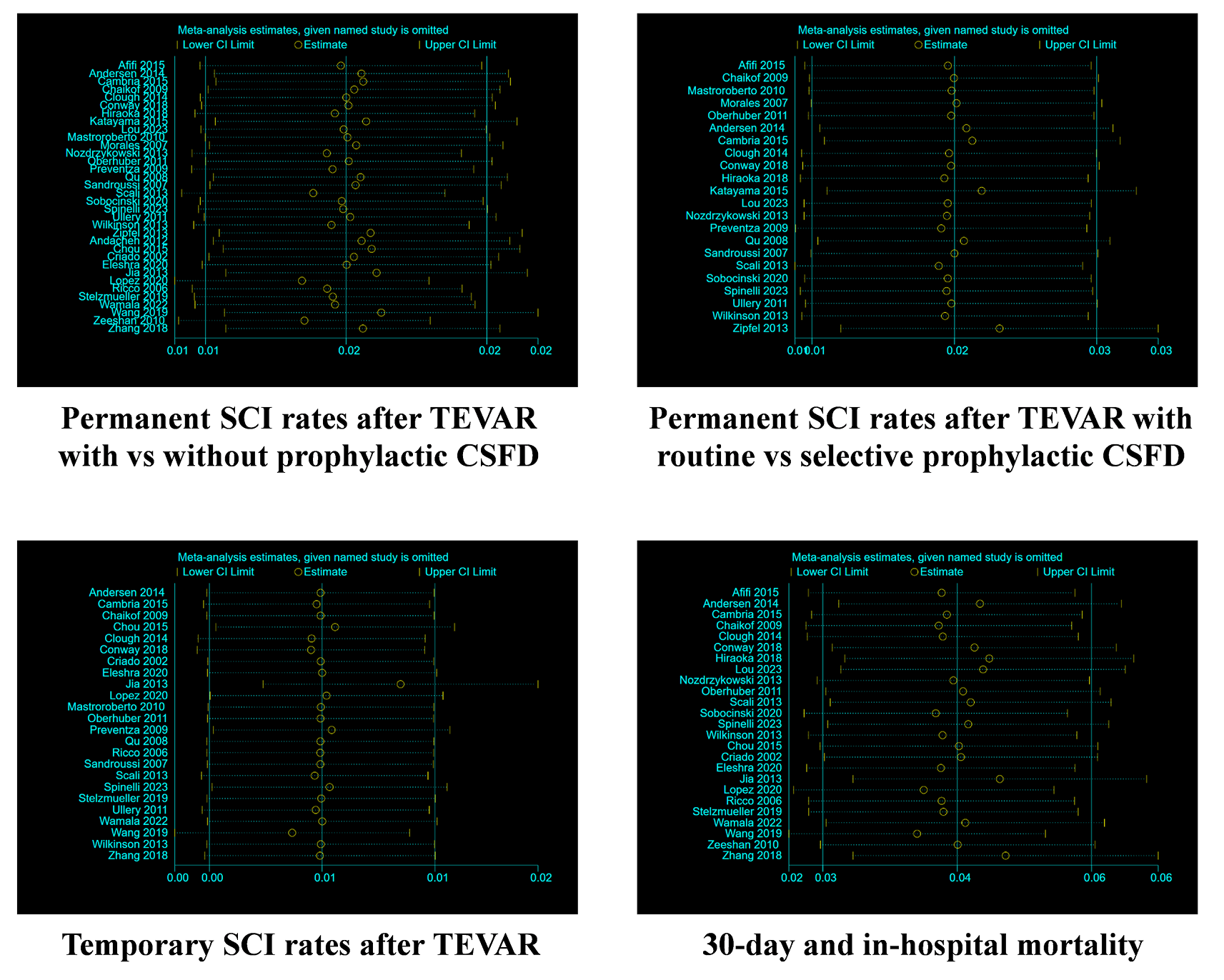

Supplement: Supplementary file 2 — Supplementary Fig. 1. Sensitivity analyses for all outcome measures. CSFD, Cerebrospinal fluid drainage; SCI, spinal cord ischemia; TEVAR, thoracic endovascular aortic repair [file 13019_2024_2603_MOESM2_ESM.tif]

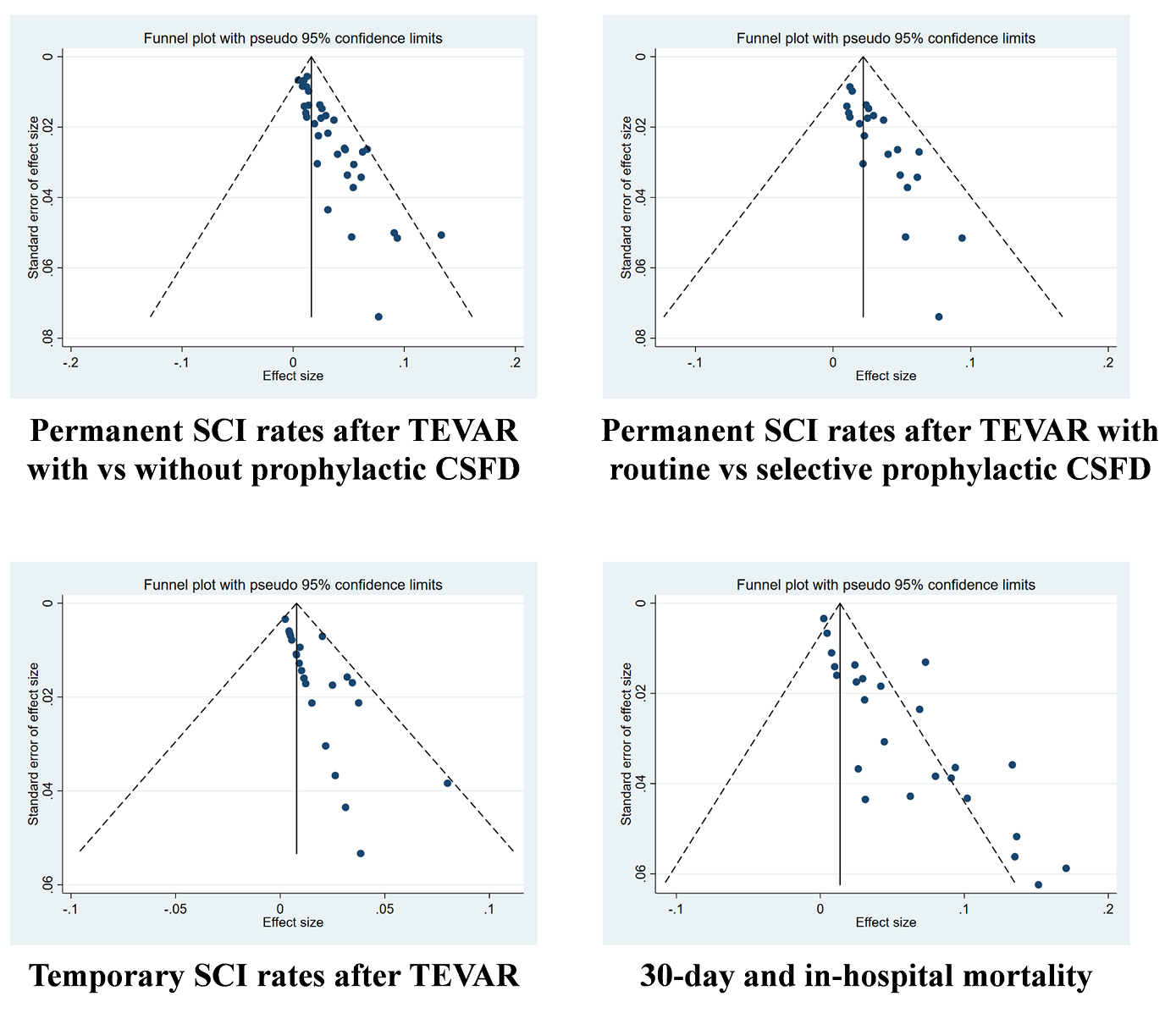

Supplement: Supplementary file 3 — Supplementary Fig. 2. Funnel plots for all outcome measures. CSFD, Cerebrospinal fluid drainage; SCI, spinal cord ischemia; TEVAR, thoracic endovascular aortic repair [file 13019_2024_2603_MOESM3_ESM.tif]

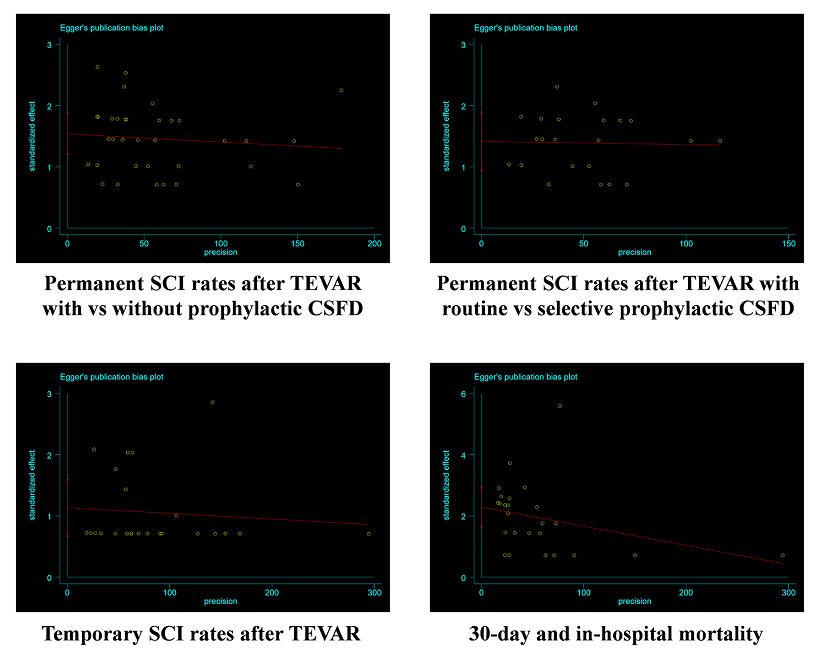

Supplement: Supplementary file 4 — Supplementary Fig. 3. Egger test for all outcome measures. CSFD, Cerebrospinal fluid drainage; SCI, spinal cord ischemia; TEVAR, thoracic endovascular aortic repair [file 13019_2024_2603_MOESM4_ESM.tif]
