# Supplementary material for: Systematic review of the effect of cerebrospinal fluid drainage on outcomes after endovascular type B aortic dissection repair
Source: J Cardiothorac Surg. 2024 Mar 12;19:116. doi: 10.1186/s13019-024-02603-3 (PMC10935911; doi:10.1186/s13019-024-02603-3)
Supplement: Supplementary file 5 — Supplementary Material 5 [file 13019_2024_2603_MOESM5_ESM.docx]

| Supplementary table 1. Summary of the included studies | | | | | | | | | | | | | | |  |
| --- | --- | --- | --- | --- | --- | --- | --- | --- | --- | --- | --- | --- | --- | --- | --- |
| Study | **Study type** | **Study period** | **Location** | **CSFD policy** | **Indications for selective CSFD** | **CSFD duration, hours** | **Other neuroprotection methods** | **Total  patients** | **Total SCI, No. (%)** | **Permanent SCI, No. (%)** | **temporary SCI, No. (%)** | **30-day/in-hospital mortality** | **CSFD-related complications** | **Downs and Black score** |  |
| Afifi 2015^[1]^ | Retrospective, single center | 2001-2014 | United States | Routine | NR | 48 | MAP maintenance | 37 | 2 (5.4) | 2 (5.4) | NR | 5 (1.4) | NR | 6 |  |
| Andacheh 2012^[2]^ | Prospective, single center | 2002-2010 | United States | Without | NR | None | None | 72 | 1 (1.4) | 1 (1.4) | NR | NR | NR | 8 |  |
| Andersen 2014^[3]^ | Retrospective, single center | 2005-2013 | United States | Selective | Intercostal-lumbar artery that supplies Adamkiewicz artery had been covered by TEVAR | 48 | Early pelvic and limb reperfusion, MEP, MAP maintenance | 44 | 0 | 0 | 0 | 0 | Intracranial hypotension | 8 |  |
| Cambria 2015^[4]^ | Prospective, multicenter | 2010-2012 | United States | Selective | NR | 48 | LSA revascularization, MAP maintenance | 50 | 4 (8.0) | 0 | 4 | 4 (8.0) | NR | 10 |  |
| Chaikof 2009^[5]^ | Retrospective, single center | 1998-2007 | United States | Routine | NR | None | None | 44 | 1 (2.2) | 1 (2.3) | 0 | 6 (13.6) | Headache | 6 |  |
| Chou 2015^[6]^ | Retrospective, single center | 2003-2009 | Taiwan | Without | NR | None | None | 119 | 1 (0.8) | 1 (0.8) | 0 | 5 (4.2) | NR | 8 |  |
| Clough 2014^[7]^ | Retrospective, single center | 2000-2014 | United Kingdom | Selective | Coverage of area that included Adamkiewicz artery | 48 | LSA revascularization | 116 | 7 (6.0) | 3 (2.6) | 4 (3.4) | 8 (6.9) | Epidural hematoma | 10 |  |
| Conway 2018^[8]^ | Retrospective, single center | 2010-2015 | United States | Selective | NR | None | None | 125 | 7 (5.6) | 3 (2.4) | 4 (3.2) | 3 (2.4) | Intracranial hypotension; Headache | 11 |  |
| Criado 2002^[9]^ | Retrospective, single center | 1999-2002 | United States | Without | NR | None | None | 16 | 0 | 0 | 0 | 0 | NR | 5 |  |
| Eleshra 2020^[10]^ | Retrospective, single center | 2010-2017 | Germany | Without | NR | None | None | 64 | 2 (3.1) | 2 (3.1) | 0 | 6 (9.3) | NR | 5 |  |
| Hiraoka 2018^[11]^ | Retrospective, single center | 2008-2014 | Japan | Selective | Aortic coverage more than 9 segments; coverage of T8 - T12 levels | 48 | MAP maintenance | 64 | 3 (4.7) | 3 (4.7) | NR | 0 | Epidural hematoma | 9 |  |
| Jia 2013^[12]^ | Prospective, multicenter | 2007-2010 | China | Without | NR | None | None | 208 | 2 (1.0) | 2 (1.0) | 0 | 0 | NR | 11 |  |
| Katayama 2015^[13]^ | Retrospective, single center | 1997-2011 | Japan | Selective | NR | None | None | 144 | 2 (1.4) | 2 (1.4) | NR | NR | Headache | 12 |  |
| Lopez 2020^[14]^ | Retrospective, multicenter | 2012-2016 | Spain | Without | NR | None | None | 90 | 6 (6.7) | 6 (6.7) | 0 | 12 (13.3) | NR | 8 |  |
| Lou 2023^[15]^ | Retrospective, single center | 2012-2020 | United States | Selective | Coverage of more than 4 segments between T8 and L1 | 48 | Early pelvic and limb reperfusion, MAP maintenance, LSA revascularization | 50 | 2 (4.0) | 2 (4.0) | NR | 0 | Subarachnoid hemorrhage | 8 |  |
| Mastroroberto 2010^[16]^ | Retrospective, single center | 2001-2008 | Italy | Routine | NR | ≥ 12 | MEP, MAP maintenance | 13 | 1 (7.7) | 1 (7.7) | 0 | NR | NR | 9 |  |
| Morales 2007^[17]^ | Retrospective, single center | 1997-2006 | United Kingdom | Routine | NR | None | None | 52 | 1 (1.9) | 1 (1.9) | NR | NR | Headache | 11 |  |
| Nozdrzykowski 2013^[18]^ | Retrospective, single center | 2000-2010 | Germany | Selective | NR | 48 | MAP maintenance | 32 | 3 (9.4) | 3 (9.4) | NR | 2 (6.3) | NR | 8 |  |
| Oberhuber 2011^[19]^ | Retrospective, single center | 1999-2011 | Germany | Routine | NR | 36 | MAP maintenance | 19 | 1 (5.3) | 1 (5.3) | 0 | 0 | NR | 6 |  |
| Preventza 2009^[20]^ | Prospective, single center | 2000-2008 | United States | Selective | Prior AAA repair | 48 | LSA revascularization | 109 | 4 (3.7) | 4 (3.7) | 0 | NR | Subarachnoid hemorrhage | 9 |  |
| Qu 2008^[21]^ | Retrospective, single center | 2005-2007 | China | Selective | Prior aortic repair; supra-aortic vessel debranching; coverage of longsegment aorta | None | None | 41 | 0 | 0 | 0 | NR | NR | 11 |  |
| Ricco 2006^[22]^ | Retrospective, multicenter | 1999-2001 | France | Without | NR | None | None | 33 | 3 (9.0) | 3 (9.0) | 0 | 5 (15.2) | NR | 10 |  |
| Sandroussi 2007^[23]^ | Retrospective, single center | 1995-2005 | United Kingdom | Selective | Coverage of longsegment aorta | 48 | MAP maintenance | 23 | 0 | 0 | 0 | NR | Intracranial hypotension | 7 |  |
| Scali 2013^[24]^ | Retrospective, single center | 2004-2011 | United States | Selective | NR | None | None | 80 | 8 (10.0) | 5 (6.3) | 3 (3.7) | 2 (2.5) | NR | 11 |  |
| Sobocinski 2020^[25]^ | Retrospective, multicenter | 2005-2015 | United States | Selective | Coverage of T8-L2 levels | 48 | MAP maintenance | 41 | 2 (4.9) | 2 (4.9) | NR | 7 (17.1) | NR | 9 |  |
| Spinelli 2023^[26]^ | Prospective, multicenter | 2010-2016 | Italy | Selective | NR | 48 | MAP maintenance | 102 | 3 (2.9) | 3 (2.9) | 0 | 3 (2.9) | Intracranial hypotension | 10 |  |
| Stelzmueller 2019^[27]^ | Retrospective, single center | 2001-2016 | Austria | Without | NR | None | None | 55 | 3 (5.5) | 3 (5.5) | 0 | 5 (9.1) | NR | 12 |  |
| Ullery 2011^[28]^ | Retrospective, single center | 2002-2010 | United States | Selective | Prior AAA repair; bilateral hypogastric artery occlusion; coverage of long-segment aorta; left subclavian artery coverage without revascularization | 48 | SSEP, MAP maintenance | 80 | 4 (5.0) | 2 (2.5) | 2 (2.5) | NR | NR | 8 |  |
| Wamala 2022^[29]^ | Retrospective, single center | 2009-2019 | Germany | Without | NR | None | None | 65 | 3 (4.6) | 3 (4.6) | 0 | 2 (3.1) | NR | 9 |  |
| Wang 2019^[30]^ | Retrospective, multicenter | 2013-2016 | United States | Without | NR | None | None | 397 | 13 (3.3) | 5 (1.3) | 8 (2.0) | 29 (7.3) | NR | 8 |  |
| Wilkinson 2013^[31]^ | Retrospective, single center | 1995-2012 | United States | Selective | NR | 48 | MAP maintenance | 49 | 3 (6.1) | 3 (6.1) | 0 | 5 (10.2) | NR | 5 |  |
| Zeeshan 2010^[32]^ | Retrospective, single center | 2002-2010 | United States | Without | NR | None | None | 45 | 6 (13.3) | 6 (13.3) | NR | 2 (4.4) | NR | 5 |  |
| Zhang 2018^[33]^ | Retrospective, multicenter | 2013-2018 | China | Without | NR | None | None | 106 | 1 (0.9) | 0 | 1 (0.9) | 0 | NR | 11 |  |
| Zipfel 2013^[34]^ | Prospective, single center | 2000-2010 | Germany | Selective | Lengthy stent-graft (200 mm) implantation with distal landing in zone 8; implantation of thoracoabdominal branched stentgrafts | 48 | LSA revascularization, MAP maintenance | 164 | 2 (1.2) | 2 (1.2) | NR | NR | NR | 12 |  |
| CSF, cerebrospinal fluid; CSFD, cerebrospinal fluid drainage; LSA, left subclavian artery; MAP, mean arterial pressure; MEP, motor-evoked potentials; NR, not reported; SCI, spinal cord ischemia; SSEP, somatosensory-evoked potentials; TEVAR, thoracic endovascular aortic repair. | | | | | | | | | | | | | | |  |
|  |  |  |  |  |  |  |  |  |  |  |  |  |  |  |  |

**References**

1. Afifi RO, Sandhu HK, Leake SS, Boutrous ML, Kumar VR, Azizzadeh A, et al. Outcomes of Patients With Acute Type B (DeBakey III) Aortic Dissection: A 13-Year, Single-Center Experience. **Circulation** **2015**, 132(8): 748-754.

2. Andacheh ID, Donayre C, Othman F, Walot I, Kopchok G, White R. Patient outcomes and thoracic aortic volume and morphologic changes following thoracic endovascular aortic repair in patients with complicated chronic type B aortic dissection. **J Vasc Surg** **2012**, 56(3): 644-650, 650.

3. Andersen ND, Keenan JE, Ganapathi AM, Gaca JG, Mccann RL, Hughes GC. Current management and outcome of chronic type B aortic dissection: results with open and endovascular repair since the advent of thoracic endografting. **Ann Cardiothorac Surg** **2014**, 3(3): 264-274.

4. Cambria RP, Conrad MF, Matsumoto AH, Fillinger M, Pochettino A, Carvalho S, et al. Multicenter clinical trial of the conformable stent graft for the treatment of acute, complicated type B dissection. **J Vasc Surg** **2015**, 62(2): 271-278.

5. Chaikof EL, Mutrie C, Kasirajan K, Milner R, Chen EP, Veeraswamy RK, et al. Endovascular repair for diverse pathologies of the thoracic aorta: an initial decade of experience. **J Am Coll Surg** **2009**, 208(5): 802-816, 816-818.

6. Chou HP, Chang HT, Chen CK, Shih CC, Sung SH, Chen TJ, et al. Outcome comparison between thoracic endovascular and open repair for type B aortic dissection: A population-based longitudinal study. **J Chin Med Assoc** **2015**, 78(4): 241-248.

7. Clough RE, Patel AS, Lyons OT, Bell RE, Zayed HA, Carrell TW, et al. Pathology specific early outcome after thoracic endovascular aortic repair. **Eur J Vasc Endovasc Surg** **2014**, 48(3): 268-275.

8. Conway AM, Qato K, Mondry LR, Stoffels GJ, Giangola G, Carroccio A. Outcomes of thoracic endovascular aortic repair for chronic aortic dissections. **J Vasc Surg** **2018**, 67(5): 1345-1352.

9. Criado FJ, Clark NS, Barnatan MF. Stent graft repair in the aortic arch and descending thoracic aorta: a 4-year experience. **J Vasc Surg** **2002**, 36(6): 1121-1128.

10. Eleshra A, Kolbel T, Panuccio G, Rohlffs F, Debus ES, Tsilimparis N. Endovascular Therapy for Nonischemic vs Ischemic Complicated Acute Type B Aortic Dissection. **J Endovasc Ther** **2020**, 27(1): 145-152.

11. Hiraoka T, Komiya T, Tsuneyoshi H, Shimamoto T. Risk factors for spinal cord ischaemia after thoracic endovascular aortic repair. **Interact Cardiovasc Thorac Surg** **2018**, 27(1): 54-59.

12. Jia X, Guo W, Li TX, Guan S, Yang RM, Liu XP, et al. The results of stent graft versus medication therapy for chronic type B dissection. **J Vasc Surg** **2013**, 57(2): 406-414.

13. Katayama K, Uchida N, Katayama A, Takahashi S, Takasaki T, Kurosaki T, et al. Multiple factors predict the risk of spinal cord injury after the frozen elephant trunk technique for extended thoracic aortic disease. **Eur J Cardiothorac Surg** **2015**, 47(4): 616-620.

14. Lopez EC, Linares PJ, Dominguez GJ, Iborra OE, Lozano VP, Solanich VT, et al. Endovascular Treatment of Descending Thoracic Aortic Pathology: Results of the Regis-TEVAR Study. **Ann Vasc Surg** **2020**, 67: 306-315.

15. Lou X, Chen EP, Duwayri YM, Jordan WD, Keeling WB, Leshnower BG. Early Results of Thoracic Endovascular Aortic Repair for the Management of Acute Uncomplicated Type B Aortic Dissection. **Semin Thorac Cardiovasc Surg** **2023**, 35(2): 289-297.

16. Mastroroberto P, Onorati F, Zofrea S, Renzulli A, Indolfi C. Outcome of open and endovascular repair in acute type B aortic dissection: a retrospective and observational study. **J Cardiothorac Surg** **2010**, 5: 23.

17. Morales JP, Taylor PR, Bell RE, Chan YC, Sabharwal T, Carrell TW, et al. Neurological complications following endoluminal repair of thoracic aortic disease. **Cardiovasc Intervent Radiol** **2007**, 30(5): 833-839.

18. Nozdrzykowski M, Etz CD, Luehr M, Garbade J, Misfeld M, Borger MA, et al. Optimal treatment for patients with chronic Stanford type B aortic dissection: endovascularly, surgically or both? **Eur J Cardiothorac Surg** **2013**, 44(3): e165-e174, e174.

19. Oberhuber A, Winkle P, Schelzig H, Orend KH, Muehling BM. Technical and clinical success after endovascular therapy for chronic type B aortic dissections. **J Vasc Surg** **2011**, 54(5): 1303-1309.

20. Preventza O, Wheatley GR, Williams J, Ramaiah V, Rodriguez-Lopez J, Diethrich EB. Identifying paraplegia risk associated with thoracic endografting. **Asian Cardiovasc Thorac Ann** **2009**, 17(6): 568-572.

21. Qu L, Raithel D. Two-year single-center experience with thoracic endovascular aortic repair using the EndoFit thoracic stent-graft. **J Endovasc Ther** **2008**, 15(5): 530-538.

22. Ricco JB, Cau J, Marchand C, Marty M, Rodde-Dunet MH, Fender P, et al. Stent-graft repair for thoracic aortic disease: results of an independent nationwide study in France from 1999 to 2001. **J Thorac Cardiovasc Surg** **2006**, 131(1): 131-137.

23. Sandroussi C, Waltham M, Hughes CF, May J, Harris JP, Stephen MS, et al. Endovascular grafting of the thoracic aorta, an evolving therapy: ten-year experience in a single centre. **Anz J Surg** **2007**, 77(11): 974-980.

24. Scali ST, Feezor RJ, Chang CK, Stone DH, Hess PJ, Martin TD, et al. Efficacy of thoracic endovascular stent repair for chronic type B aortic dissection with aneurysmal degeneration. **J Vasc Surg** **2013**, 58(1): 10-17.

25. Sobocinski J, Dias NV, Hongku K, Lombardi JV, Zhou Q, Saunders AT, et al. Thoracic endovascular aortic repair with stent grafts alone or with a composite device design in patients with acute type B aortic dissection in the setting of malperfusion. **J Vasc Surg** **2020**, 71(2): 400-407.

26. Spinelli D, Weaver FA, Azizzadeh A, Magee GA, Piffaretti G, Benedetto F, et al. Endovascular treatment of complicated versus uncomplicated acute type B aortic dissection. **J Thorac Cardiovasc Surg** **2023**, 165(1): 4-13.

27. Stelzmueller ME, Nolz R, Mahr S, Beitzke D, Wolf F, Funovics M, et al. Thoracic endovascular repair for acute complicated type B aortic dissections. **J Vasc Surg** **2019**, 69(2): 318-326.

28. Ullery BW, Cheung AT, Fairman RM, Jackson BM, Woo EY, Bavaria J, et al. Risk factors, outcomes, and clinical manifestations of spinal cord ischemia following thoracic endovascular aortic repair. **J Vasc Surg** **2011**, 54(3): 677-684.

29. Wamala I, Nazari-Shafti M, Heck R, Penkalla A, Montagner M, Staffa SJ, et al. Aortic remodelling and late outcomes following thoracic endovascular repair with a bare-metal stent distal extension among patients with complicated type-B aortic dissection. **Interact Cardiovasc Thorac Surg** **2022**, 35(5).

30. Wang GJ, Cambria RP, Lombardi JV, Azizzadeh A, White RA, Abel DB, et al. Thirty-day outcomes from the Society for Vascular Surgery Vascular Quality Initiative thoracic endovascular aortic repair for type B dissection project. **J Vasc Surg** **2019**, 69(3): 680-691.

31. Wilkinson DA, Patel HJ, Williams DM, Dasika NL, Deeb GM. Early open and endovascular thoracic aortic repair for complicated type B aortic dissection. **Ann Thorac Surg** **2013**, 96(1): 23-30, 230.

32. Zeeshan A, Woo EY, Bavaria JE, Fairman RM, Desai ND, Pochettino A, et al. Thoracic endovascular aortic repair for acute complicated type B aortic dissection: superiority relative to conventional open surgical and medical therapy. **J Thorac Cardiovasc Surg** **2010**, 140(6 Suppl): S109-S115, S142-S146.

33. Zhang H, Qiao T. Aortic remodeling in Type B aortic dissection after thoracic endovascular aortic repair with an aortic extender cuff implantation. **Clin Interv Aging** **2018**, 13: 2359-2366.

34. Zipfel B, Buz S, Redlin M, Hullmeine D, Hammerschmidt R, Hetzer R. Spinal cord ischemia after thoracic stent-grafting: causes apart from intercostal artery coverage. **Ann Thorac Surg** **2013**, 96(1): 31-38.
